# Sleep deprivation increases the regularity of isometric torque fluctuations

**DOI:** 10.1007/s00221-024-06810-1

**Published:** 2024-03-07

**Authors:** João H. Oliveira, Paulo Santos, Pedro Pezarat-Correia, João R. Vaz

**Affiliations:** 1 Neuromuscular Research Lab, Faculty of Human Kinetics, University of Lisbon, Lisbon, Portugal; 2 CIPER,Faculty of Human Kinetics, University of Lisbon, Lisbon, Portugal; 3 Egas Moniz Center for Interdisciplinary Research (CiiEM), Egas Moniz School of Health & Science, Almada, Portugal

**Keywords:** Complexity, Variability, Entropy, Force, Motor control, Sleep

## Abstract

The regularity of the fluctuations present in torque signals represent the adaptability of the motor control. While previous research showed how it is affected by neuromuscular fatigue and ageing, the underlying mechanisms remain unclear. It is currently under debate whether these changes are explained by central or peripheral neuromuscular mechanisms. Here, we experimentally manipulated the sleep of thirteen young adults through a supervised 24 h-sleep deprivation protocol. This study aimed to investigate the effect of sleep deprivation on the regularity of torque fluctuations, and other standard torque-related outcomes (Peak Torque – PT – and Rate of Torque Development – RTD). The participants were asked to perform knee extension maximal voluntary contractions (MVC) and submaximal knee extensions at 40% of MVC for 30 s. PT and RTD were calculated from the MVC and the regularity of the torque fluctuations was determined on the submaximal task through Sample Entropy (SampEn). In addition, rate of perceived effort (RPE) was collected. We found no significant changes in PT and RTD. The regularity of torque fluctuations significantly increased (i.e., a decrease in SampEn) after 24 h-sleep deprivation (PRE = 1.76 ± 0.268, POS24 = 1.71 ± 0.306; *p* = 0.044). Importantly, we found a negative correlation between RPE and SampEn relative changes after sleep deprivation. This study brings new insights towards the understanding of the underlying mechanisms that explain changes in torque fluctuations, demonstrating that these changes are not limited to neuromuscular processes but are also likely to be affected by other domains, such as psychological profile, which can indirectly affect the neural drive to the muscles.

## Introduction

Sleep has been shown to be critical for physiological and cognitive functioning (Fullagar et al. 2015; Knowles et al. 2018). Its restorative effects on immune system, endocrine function and cognitive performance make sleep essential for human health (Souissi et al. 2020) with significant impact on physical development, emotional regulation, cognitive performance, and quality of life (Watson 2017). However, despite the recognized importance of sleep, people tend to experience inadequate sleep that can occur as a result of sleep deprivation, i.e., a sustained state of wakefulness with no sleep, or sleep restriction, i.e., a chronically reduced sleep duration (Knowles et al. 2018).

Although the effect of inadequate sleep on health, well-being and work productivity is well documented in scientific literature (Chaput et al. 2018; Chattu et al. 2018; Grandner 2017; Walker 2019; Watson et al. 2015a, b), less and more conflicting evidence exists for its effect on exercise performance. A recent review (Fullagar et al. 2015) concluded that athletic performance is likely impaired by sleep deprivation, despite the extent and nature of this impairment still unclear, partly due to differences in experimental designs, such as the duration of the sleep deprivation protocols, and the muscle group or type of muscle contraction (isometric, isokinetic, dynamic) tested in each study. Particularly, in relation to muscle strength, sleep-deprivation related research suggests that sleep-deprived individuals typically exhibit a decreased performance in submaximal tasks and are less affected in maximal type of motor tasks, although conflicting results exist in the literature (Fullagar et al. 2015; Watson 2017). Importantly, the decrease in performance has previously been attributed to the increased perceived exertion after sleep deprivation (Cullen et al. 2019; Fullagar et al. 2015; Oliver et al. 2009; Roberts et al. 2019). However, although several studies examined the effect of sleep deprivation on neuromuscular performance, its effect on torque control has not yet been investigated.

Torque control consists on how the neuromuscular system regulates the torque production through multiple mechanisms such as motor unit recruitment, motor unit firing rates, motor unit synchronization, feedback loops, and attentional control, that dynamically interact to generate and sustain a targeted torque level (Vaillancourt and Newell 2002). Typically, torque control is assessed through targeted submaximal isometric or isotonic contractions at a percentage of maximal voluntary contraction (Enoka and Farina 2021), during which the torque produced is not constant, but exhibits fluctuations that are believed to contain hidden information about the neural mechanisms underlying torque control instead of being considered unwanted noise as in the past (Stergiou and Decker 2011).

Traditionally, these fluctuations have been quantified according to their magnitude, using either absolute - standard deviation (SD) - or relative (i.e., normalized to the mean) measures - coefficient of variation (CV) (Enoka et al. 2003; Enoka and Farina 2021). However, the fluctuations present in the torque output are known to be characterized by a non-regular temporal structure and exhibit complex patterns (Slifkin and Newell 1999). While magnitude-based measures represent the torque steadiness, complexity-based measures represent the adaptability of the neuromuscular system, that is, the ability to adapt motor output to the task demands (Vaillancourt and Newell 2003). Interestingly, Stergiou et al. (2006) proposed the optimal human movement variability model which proposes that healthy biological systems are characterized by an optimal amount of variability, defined by a highly complex, chaotic, structure. According to this model, that relates in an inverted U-shape relationship the concepts of regularity and complexity, this optimal complexity characterizes systems with increased motor solutions, i.e., with greater capacity to adapt to changes in the environment, while the loss of complexity indicates a more rigid or more unstable control of the system which becomes less adaptable to perturbations in the environment.

Torque complexity is, therefore, an important measure of motor control which has recently been proposed as an indirect index of the functional capacity of the neuromuscular system (Pethick et al. 2016, 2018b, c). Specifically, low levels of torque complexity reflect a neuromuscular system with reduced number of motor solutions and, therefore, less capable of adapting the motor output to match the task demands. Moreover, Mear et al. (2023) demonstrated that the dynamic balance performance can be explained by changes in torque complexity measures, indicating the capacity of torque complexity to reflect adaptability and providing a parallel with previous research on magnitude-based measures (torque steadiness) and walking performance (Davis et al. 2020), the risk of falls in older adults (Carville et al. 2007) and postural sway during upright standing (Davis et al. 2020). Interestingly, Ravi et al. (2021) demonstrated that the capacity to recover after a perturbation during the locomotion was faster when the individuals walk synchronised to a complex auditory stimulus (i.e. auditory stimulus incorporating optimal complexity in its temporal structure) than to a rigid auditory stimulus (i.e. auditory stimulus without complexity in its temporal structure), suggesting greater adaptability and hence reflecting the relevance of the optimal complexity to the individual’s functional capacity.

Furthermore, the loss of torque complexity has been associated with the ageing process (Fiogbé et al. 2018; Vaillancourt and Newell 2003), pathology (Vaillancourt and Newell 2002) and neuromuscular fatigue (Oliveira et al. 2022; Pethick et al. 2015, 2016, 2018b, c, 2019a, b, 2021).

Neuromuscular fatigue has been shown to alter torque complexity by altering the temporal structure of its fluctuations. Specifically, these fluctuations become more regular and predictable, resulting in a less complex output and, therefore, in a decreased adaptability of the neuromuscular system with consequences at the motor control level, namely the increased risk of failing a motor task, such as correcting a fall or producing a required joint torque (Pethick et al. 2018b). Pethick et al. (2018a, 2019a, b, 2021) suggested that the fatigue-induced loss of torque complexity is an integrated response to both peripheral and central processes. Interestingly, Pethick et al. (2018a) demonstrated that the ingestion of caffeine, a supplement used to increase physiological alertness and cognitive performance, attenuates the effects of fatigue on torque complexity. If central processes play an essential role in torque regulation and based on its potential clinical value and on this interesting finding related to the attenuation of complexity loss after caffeine ingestion, understanding how sleep deprivation, a prominent problem in modern society, could affect torque complexity would likely provide more information on this research area.

Therefore, we aimed to investigate if a one night of sleep deprivation would affect torque-related measures, with a primary interest in the temporal structure of torque’s fluctuations (i.e., torque complexity). We hypothesised that one night of sleep deprivation would increase the regularity of torque fluctuations (i.e., decreased torque complexity). We also aimed to investigate the effect of sleep deprivation in the magnitude of torque variability, measured through coefficient of variation. Considering the literature that investigated the effect of neuromuscular fatigue in the coefficient of variation (Pethick et al. 2018a, 2019a, b), we hypothesised an increase in coefficient of variation with one night of sleep deprivation. In addition, we aimed to assess the sleep deprivation effects on standard torque-related measures, such as peak torque and the rate of torque development, obtained during Maximal Voluntary Isometric Contractions (MVIC). Considering the maximal nature of such a task and based on previous findings suggesting that maximal motor tasks are not affected by sleep deprivation, we hypothesised no changes in both measures. Lastly, we aimed to examine the effect of sleep deprivation on rate of perceived exertion and its association with the relative changes of torque measures. Considering that perceived exertion has been shown to increase with sleep deprivation (Cullen et al. 2019; Fullagar et al. 2015; Oliver et al. 2009; Roberts et al. 2019), we hypothesised an increase in rate of perceived exertion with one night of sleep deprivation. Additionally, considering the previously mentioned literature, we also hypothesised a negative correlation between rate of perceived exertion and torque complexity relative changes, and a positive correlation between rate of perceived exertion and coefficient of variation relative changes.

## Methods

### Participants

Thirteen young healthy and active male volunteers (age:24.2 ± 2.45; height: 1.74 ± 0.06 m; body mass: 69.8 ± 8.07 kg; body mass index: 23.0 ± 1.67 kg/m^2^) participated in this study. Participants were free of any neurological disease, known musculoskeletal disorder, cardiovascular and respiratory disorders. Additionally, those with a lower limb injury six months before the experiment were excluded. Finally, participants with poor sleep quality were excluded. Sleep quality was assessed through the Pittsburgh Sleep Quality Index (PSQI) questionnaire (Del Rio João et al. 2017), and those that exhibited a PSQI score > 5 were considered to have poor sleep quality and, therefore, did not participate in this study. Prior to participation, the participants signed an informed consent previously approved by the Ethics Committee of the Faculty of Human Kinetics (approval number #02/2020) and all procedures adhered to the Declaration of Helsinki.

### Experimental design

This study followed a one-group pretest-posttest pre-experimental design. Participants were asked to visit the laboratory for three different sessions (FAM, PRE and POS). During their participation, they were instructed to maintain their typical sleep patterns. They were also instructed to abstain from alcohol, caffeine, cacao, tea and other stimulant substances from 8 to 12 h before the day the sleep deprivation protocol occurred until the end of their participation in the study. Furthermore, the participants were also instructed to avoid moderate to vigorous physical activity and resistance training until the completion of the study. They were asked to fulfil the Horne & Ostberg Morningness-Eveningness Questionnaire (MEQ) – Portuguese version (Silva et al. 2002) to determine their chronotype.

During their first visit, the participants were familiarised (FAM) with all testing equipment and procedures. The second visit (PRE) to the laboratory occurred approximately seven days after the FAM session. Then, participants were instructed not to sleep during the day and were asked to return to the laboratory on the same day (~ 10pm) and remain under the supervision of a researcher until the POS session was completed. Sedentary and light activities such as reading, watching television, listening to music, playing video games, standing up and walking were allowed at the night of sleep deprivation (Arnal et al. 2016; Temesi et al. 2013), although the screen-related activities were limited.

The three sessions were always conducted at the same time of the day (7 am–9 am) to avoid daily variations of muscle force related to human circadian rhythms (Araujo et al. 2011). In each session, participants were asked to perform maximal and submaximal knee extension tests, as described below in detail.

### Data collection

Participants were seated in the chair of a Biodex System 3 Pro isokinetic dynamometer (Biodex Medical System 3, Shirley, NY), initialised and calibrated according to the manufacturer’s instructions, with their dominant leg attached to the lever arm of the dynamometer and the seating position adjusted to ensure that the lateral epicondyle of the femur was aligned with the axis of rotation of the lever arm. The relative hip and knee angles were set at 90° to adjust the position of isokinetic dynamometer. Before testing, participants performed a range of submaximal isometric knee extensions to ensure proper familiarisation with the testing task and to ensure warm-up. All measures were taken at 70° of knee flexion (full knee extension being 0°), and participants were asked not to alter their posture and focus on the task. The lower leg was attached to the lever arm above the malleoli with a velcro strap. Straps were secured firmly across both shoulders and the waist to prevent extraneous movement and the use of the hip extensors during the contractions. The seating position was recorded during the first visit and replicated for each subsequent visit.

During the Maximal Voluntary Isometric Contraction (MVIC) tasks, the participants were asked to perform three isometric contractions lasting 5 s each with a 60-second interval of rest between trials. They were instructed to exert their maximum force as fast and hard as possible with verbal encouragement and visual feedback from the researcher and the dynamometer software, respectively. For the submaximal trials, participants performed a sustained contraction at a target torque of 40% of MVIC. This torque intensity was defined based on the MVIC from session one and used for all sessions. Participants were instructed to match their instantaneous torque with a target bar superimposed on a display in front of them and asked to continue matching this torque for 30 s. Two trials, with a 1-minute resting period in between, were conducted. Data were sampled through Biopac MP100 (Biopac Systems Inc., California, USA) interfaced with a personal computer. All signals were sampled at 1000 Hz. Data were collected in Acknowledge (Version 3.9.1. Biopac Systems, Inc., California, USA) and further exported to Matlab® (The MathWorks Inc., Natick, MA, USA). At the end of each torque protocol, the participants´ Rate of Perceived Exertion (RPE) was assessed using a Borg’s CR-10 scale modified by Foster et al. (1995).

### Data analysis

All data were analysed using code written in Matlab® R2018a (The MathWorks, Natick, MA, USA). Torque signals were first low-pass filtered (Butterworth 10 Hz, 4th order). For MVIC trials, Peak Torque (PT) and Rate of Toque Development (RTD) were determined. PT was defined as the highest torque value. RTD was calculated as the slope of the torque-time relationship in the following intervals: 0-50ms, 50-100ms and 100-150ms (RTD0-50, RTD50-100, RTD100-150, respectively). Torque onset (0ms) was defined as the time the torque was greater than 5 N.m. For statistical purposes, the highest PT and RTD values amongst the three trials were used.

Regarding the submaximal trials, the signals were first downsampled to 50 Hz. This was determined after visually inspecting the signals’ spectrograms and finding 50 Hz as the appropriate sampling frequency to contain all the relevant information. Then, the signals were cropped to remove the ascending and descending components of the isometric contractions. Therefore, the analysed signals only accounted for the time the participant matched the targeted MVIC%. Then, the magnitude of variability was obtained through the coefficient of variation (CV), and the temporal structure of variability was obtained through Sample Entropy (SampEn).

SampEn (Richman and Moorman 2000) was used to determine the temporal structure of the torque output. It determines the inverse probability that short sequences of data points are repeated throughout a temporal sequence of points. Therefore, time series with repeatable sequences of data points would result in a regular output leading to a lower SampEn value, while the absence of this repeatability of sequences of data points would result in more irregular output characterised by higher SampEn values. Thus, a perfectly repeatable time series has a SampEn value equal to zero, and a perfectly random time series has a SampEn value converging towards infinity (Richman and Moorman 2000). In this study, a pattern length (m) of 2, error tolerance (r) of 0.2 and data length (N) of 1500 data points (i.e., 50 Hz x 30 s) were selected and used in the determination of SampEn values (Raffalt et al. 2023; Yentes et al. 2013). The reliability of entropy measures was shown to be optimal when these input values are identical for all trials and participants (Cavanaugh et al. 2005). SampEn and CV were extracted from the same previously cropped signal. For statistical purposes, the average of the two trials was used.

### Statistical analysis

All statistical analyses were carried out using IBM SPSS Statistics for Windows (Version 25.0. Armonk, NY: IBM Corp). Standard descriptive statistics (mean and standard deviation) were used to summarise the data. All data were tested for normality using Shapiro-Wilk tests. One tailed paired samples *t*-test was used to determine the effects of pre- to post-24 h of sleep deprivation on submaximal torque parameters (SampEn and CV) and RPE variables. Cohen’s *d* was calculated as a measure of effect size, as 𝑑=$\frac{t}{\sqrt{N}}$, where *t* stands for the statistical value and *N* for the number of observations. A multivariate analysis of variance (MANOVA) was conducted to determine the effect of sleep deprivation on maximal torque parameters (PT and RTD). Tests for multivariate normality, homogeneity of variance matrices and linearity were performed to check the assumptions of MANOVA. Additionally, a Pearson correlation was used to examine the association between RPE and SampEn and CV relative changes after sleep deprivation. Statistical significance was set at *p* < 0.05.

## Results

### Maximal voluntary isometric contraction related parameters

MANOVA results showed no significant effect of 24 h of sleep deprivation on PT, RTD_0–50_, RTD_50–100_ and RTD_100–150_ (*F*_(4, 21)_ = 0.594, Wilk’s Λ = 0.898, *p* = 0.671, partial η^2^ = 0.102).

### Submaximal task-related parameters

**Fig. 1:**
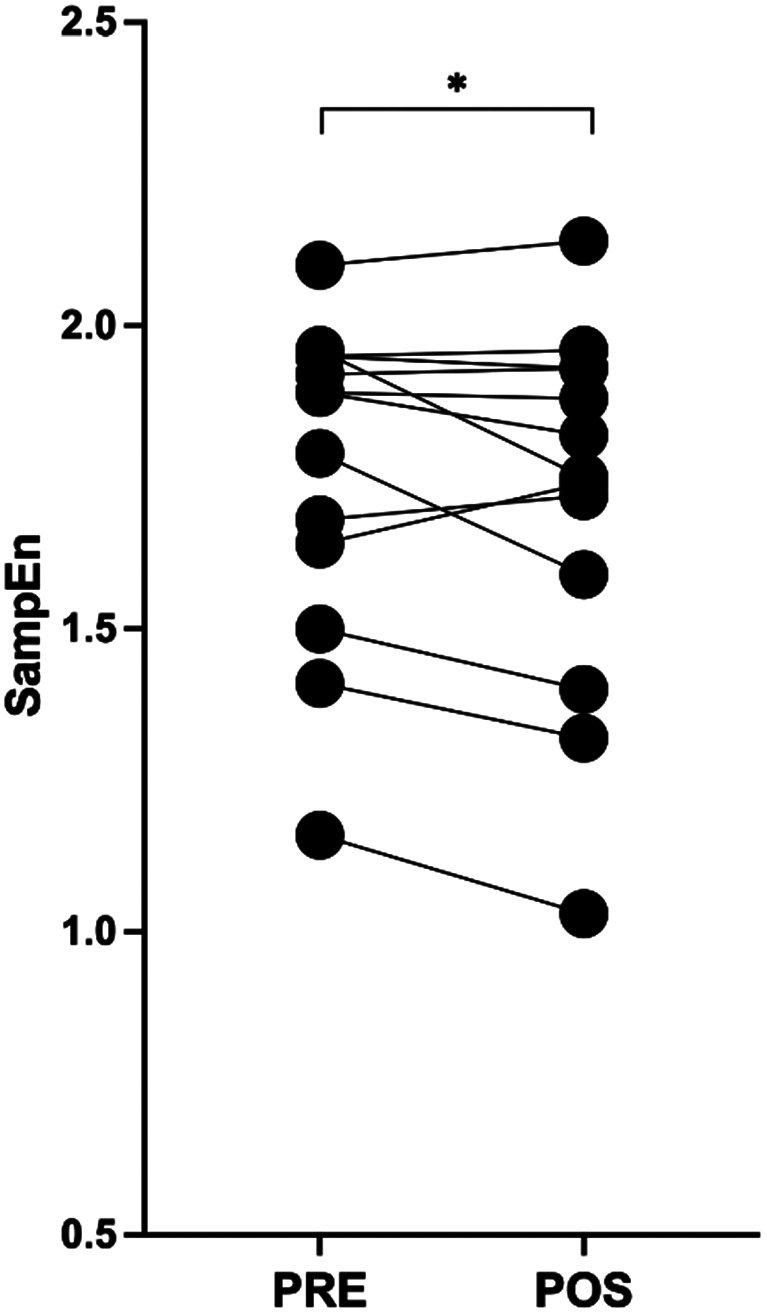
Sample Entropy pre-to-post 24 h of sleep deprivation. Each bullet pair represents a participant. * indicates significant differences between pre and post sleep deprivation at *p* < 0.05

For SampEn (Fig. 1), a significant decrease (t = 1.86, *p* = 0.044, *d* = 0.516; PRE = 1.76 ± 0.27, POS = 1.71 ± 0.31) was observed. Similarly, 24 h of sleep deprivation resulted in a significant decrease of CV (t = 2.86, *p* = 0.014, *d* = 0.794; PRE = 1.43 ± 0.36, POS = 1.27 ± 0.27).

Regarding RPE, we observed a significant increase (t=-3.46, *p* = 0.006, *d*=-1.04; PRE = 5.45 ± 1.04, POS = 6.55 ± 1.29) with sleep deprivation.

Lastly, we found that RPE and SampEn relative changes after sleep deprivation were moderately negatively correlated (*r*=−0.570; *p* = 0.034). Conversely, no correlation between RPE and CV relative changes was found (*r* = 0.244; *p* = 0.766).

## Discussion

This study investigated the effects of one night of sleep deprivation on torque-related measures. First, we hypothesised that sleep deprivation would lead to an increase of torque regularity. Our results support this hypothesis as we observed greater regularity of the torque output (i.e., lower SampEn) after one night of sleep deprivation. Additionally, we also investigated the sleep deprivation effects on peak torque and the rate of torque development. We hypothesised that sleep deprivation would affect neither. This hypothesis was supported by our results since we observed no effect of sleep deprivation on maximal torque measures. Lastly, we tested the association between perceived exertion and torque measures relative changes. As hypothesised, we observed a significant negative correlation between torque regularity and perceived exertion indicating the greater the effect of sleep deprivation on perceived effort, the greater the regularity of torque fluctuations. Conversely, contrary to our hypothesis, no correlation between coefficient of variation and perceived effort was found after sleep deprivation.

Torque complexity represents the neuromuscular system´s ability to adapt to changes in the environment and, therefore, is considered an indicator of functional capacity and adaptability (Pethick et al. 2016, 2018b, c). Recent literature suggests that the loss of torque complexity can be associated with neuromuscular fatigue (Oliveira et al. 2022), and these fatigue-related changes in torque complexity can be explained by the integrated response of central and peripheral mechanisms (Pethick et al. 2015, 2016, 2018b, c, 2019a, b, 2021). The present study expanded the investigation of potential central contribution by experimentally manipulating sleep through a sleep deprivation protocol.

Sleep deprivation negatively affects neurocognitive function and physical performance, particularly during submaximal tasks (Fullagar et al. 2015). This supports our torque complexity related findings. We observed a decrease in SampEn, which indicates a more constrained and rigid control of the neuromuscular system, resulting in a less adaptable system (i.e., less complex).

A plausible physiological explanation to the observed results refers to mental fatigue, the psychobiological state caused by the prolonged mental exertion associated with sleep deprivation. Indeed, mental fatigue has been shown to impair the performance of tasks at a submaximal intensity, and this detrimental effect has been attributed to a higher perception of effort (Pageaux and Lepers 2018; Van Cutsem et al. 2017), which aligns with our results since we observed a significant increase in RPE after sleep deprivation. Notably, we found a moderate correlation between the changes in the rate of perceived effort and the changes in torque regularity after sleep deprivation.

Interestingly, it has been suggested that brain neurotransmitter systems might be implicated in the development of mental exertion with the most important role for the effect of dopamine and adenosine in multiple brain regions such as the prefrontal cortex and the anterior cingulate cortex (Meeusen et al. 2021). It has been proposed that mental exertion promotes an accumulation of adenosine within active brain areas such as anterior cingulate cortex, a structure that is associated with the perception of effort (Martin et al. 2018; Pageaux and Lepers 2018; Pageaux et al. 2013). Martin et al. (2018) suggested that adenosine acts in two ways: by increasing perception of effort during subsequent effortful tasks, and by impairing motivation, or the willingness to exert effort, likely via an interaction with dopamine in the anterior cingulate cortex. Furthermore, the literature suggested that prolonged mental exertion can result in decreased dopamine levels, which might negatively affect effort/reward perception, resulting in the choice of low-cost behavioural alternatives or in the signalling to the need for increased attentional resources to enhance control (Lorist et al. 2009; Moeller et al. 2012). Therefore, the increased perception of effort induced by mental fatigue associated to sleep deprivation could have affected the interaction between the multiple components of the neuromuscular system, resulting in a more constrained and rigid control of torque production, reflected by lower levels of entropy.

Functional magnetic resonance imaging (fMRI) studies have been demonstrated that sleep deprivation leads to decreased brain activation in the frontoparietal attention network (prefrontal cortex and intraparietal sulcus) and in the salience network (insula and medial frontal cortex) (Ma et al. 2015; Skurvydas et al. 2020a). Since the task used to assess torque complexity consisted in the participants match their instantaneous torque with a target bar superimposed on a display in front of them for 30 s, some degree of attention was involved in the accomplishment of the task. Attention deficits caused by sleep deprivation could, therefore, have influenced the capacity of individuals to regulate the torque production. Although it cannot establish a causal relationship, it provides important insights regarding the regulation of torque complexity. An increase in rate of perceived effort, a perception thought to be involved in the engagement and the regulation of physical behaviour (Pageaux and Lepers 2018), associated to a decreased brain activation following sleep deprivation might affect how individuals control the torque production and thus explain the observed changes in temporal structure of torque fluctuations.

Importantly, the results of the present study should be interpreted with caution due to the magnitude of entropy’s changes. Indeed, despite the overall decrease in entropy, indicating an increase in torque regularity, we observed that some participants showed decreases in torque regularity, while others showed increases or no changes (Fig. 1). Therefore, future research is needed to further explore the effect of sleep deprivation in torque regularity. For instance, considering that sleep deprivation can affect individuals differently, future studies could manipulate the duration of sleep deprivation’s protocol and explore the role of individual chronobiology in explaining the results observed in torque regularity. As previously been observed, gait complexity is also slightly altered between chronotypes (Vaz et al. 2023). Moreover, studying the effect of chronic sleep restriction, a prominent problem in modern society, on torque regularity could be important to understand its effect on motor control. Likewise, given that torque regularity reflects the adaptability of the neuromuscular system to task challenges (Stergiou and Decker 2011), future studies should explore the effect of increasing the complexity of the task, for example through the addition of a cognitive task to the motor task (i.e., dual tasking paradigm).

Interestingly, and contrary to our hypothesis, we observed a decrease in CV and found no correlation between RPE and CV. This finding further supports the evidence that CV and SampEn provide distinct information. While CV provides information about the magnitude of torque variability, SampEn informs about the temporal structure of this variability representing the adaptability of the neuromuscular system (Stergiou and Decker 2011). Our results suggest that sleep deprivation increased force steadiness which could be potentially explained by the freezing of degrees of freedom in task execution as a response to sleep deprivation. That is, it seems that, after 24 h of sleep deprivation, individuals adopted a rigid control of torque production in order to accomplish the task goal, resulting in a less variable (decreased CV), and more regular torque output (decreased SampEn).

Furthermore, in the present study we found that the maximal torque parameters (PT and RTD) were not affected by 24 h of sleep deprivation. These results are in line with the literature that suggests that tasks requiring short-term high-power output (anaerobic and maximal strength performance) seem to be unaffected by sleep deprivation (Fullagar et al. 2015; Skurvydas et al. 2020a, b; Thun et al. 2015). Temesi et al. (2013) found no effect of one night of sleep deprivation on maximal voluntary isometric contraction of the knee extensors. The same was previously observed by Vaara et al. (2018) after 60 h of sleep deprivation. Recently, Skurvydas et al. (2020b) have studied the effect of two nights of sleep deprivation on motor performance and found that knee extensor’s maximal voluntary isometric contraction did not change at 24 h of sleep deprivation but did at 48 h. The authors have attributed this result, in part, to the increase in sleepiness and in the feeling of fatigue, and a significant decrease in motivation and vigour which are in line with our results. Furthermore, others have found that maximal voluntary isometric contraction can be affected by sleep deprivation (Arnal et al. 2016; Bulbulian et al. 1996; Skein et al. 2011; Takeuchi et al. 1985). These conflicting results have been attributed to differences in methodology and in research designs (sleep deprivation protocol, type of task, muscle group) as well as to the statistical power of each study (Fullagar et al. 2015; Skurvydas et al. 2020a). Compared to other studies, a major strength of the present study refers to the in-lab supervised sleep deprivation protocol which assured that the participants were in fact fully sleep deprived.

Despite these conflicting results and variations in the methodologies, the physiological mechanisms responsible for the decrease in performance after sleep deprivation remain unclear.

A potential limitation of the present study was the lack of other physiological measurements such as electromyography (EMG) which precludes further discussion about the mechanisms that lead to the observed results in both maximal and submaximal torque parameters. However, previous research observed neural aspects of the neuromuscular function (muscle activation and neural drive) to be unaffected by sleep deprivation (Arnal et al. 2016; Gonçalves et al. 2020; Temesi et al. 2013). Specifically, Temesi et al. (2013) and Arnal et al. (2016) found no differences in neuromuscular activation, suggesting that the observed performance decrement was unlikely caused by the increased central or peripheral fatigue. Instead, the authors attributed the decrease in performance to the increase in perceived exertion, which are in line with our results since we found an increase in RPE pre-to-post 24 h of sleep deprivation and a correlation between perceived exertion and relative changes in SampEn. Recently, it has been reported that the neural drive to the muscle remains preserved after 24 h of sleep deprivation (Gonçalves et al. 2020; Skurvydas et al. 2020b), but it is altered under more prolonged protocols: 30 h (Skein et al. 2011) and 48 h (Skurvydas et al. 2020b). Interestingly, Gonçalves et al. (2020) found that 24 h of sleep deprivation affects motor control through an increase of the antagonist/agonist co-activation. Moreover, the effect of sleep deprivation in common synaptic output, a phenomenon that has been considered to be determinant of the magnitude of torque fluctuations (Enoka and Farina 2021) and has been speculated to contribute to the temporal structure of torque output (Pethick et al. 2021) is yet to be established.

Thus, future studies should consider the use of EMG, particularly the high-density EMG, in order to better explain the physiological mechanisms behind the effects of sleep deprivation on maximal and submaximal torque production.

## Conclusion

The present study demonstrated that torque regularity increases with sleep deprivation providing new insights towards the understanding of possible mechanisms underlying torque temporal fluctuations. While it would be difficult to precisely understand such mechanisms in the future, since it refers to the output generated from a dynamic interaction between several elements, this study expands the current research by adding how central processes likely participate in torque regulation. Future research should aim to incorporate statistical models that provide a relative contribution of different system’s elements (e.g., motor unit recruitment, co-contraction level, perception of effort, mood, chronotype, etc.), which ultimately will allow us to understand the major contributors to such complexity loss and, therefore, can aim how to restore such capacity in populations in which sleep is affected (e.g., shift workers, athletes). Moreover, the decrease in submaximal performance could be partly explained by the effect of sleep deprivation on the individuals’ perception of effort demonstrating that the regulation of torque production is not limited to neuromuscular processes but are also likely to be affected by other domains, such as psychological profile, that can indirectly affect the neural drive to the muscles.

## Data Availability

The datasets generated during and/or analysed during the current study are available from the corresponding author on reasonable request.
